# Dual Role of Yeasts and Filamentous Fungi in Fermented Sausages

**DOI:** 10.3390/foods13162547

**Published:** 2024-08-15

**Authors:** Rossana Sidari, Rosanna Tofalo

**Affiliations:** 1 Department of Agraria, Mediterranea University of Reggio Calabria, 89122 Reggio Calabria, Italy; 2 Department of Bioscience and Technology for Food, Agriculture and Environment, University of Teramo, 64100 Teramo, Italy

**Keywords:** yeasts, filamentous fungi, fermented sausages, starter cultures

## Abstract

This contribution aims to review the presence and the potential double role—positive or beneficial and negative or harmful—of fungi in fermented sausages as well as their use as starter cultures. Traditionally, studies have been focused on lactic acid bacteria; however, over the years, interest in the study of fungi has increased. The important contribution of yeasts and filamentous fungi to the quality and safety of fermented sausages has emerged from reviewing the literature regarding these fermented products. In conclusion, this review contributes to the existing literature by considering the double role of filamentous fungi and yeasts, the global fermented sausage market size, the role and use of starters, and the starters mainly present in the worldwide market, as well as the main factors to take into account to optimize production. Finally, some suggestions for future broadening of the sector are discussed.

## 1. Introduction

Fungi are commonly able to grow in a variety of food and beverages, including meat-derived foods. Meat, in fact, is a perishable substrate that sustains microbial growth. Fermentation is an ancient method to preserve meat mainly thanks to lactic acid bacteria (LAB). Their activity modify the environment and reduces water activity, resulting in inhibiting the growth of undesirable microorganisms and in the production of a finished product with a prolonged shelf life. Besides LAB, yeasts and filamentous fungi contribute to the microbial community and play a key role in food processing thanks to their metabolic versatility. Fungal biota, in fact, is involved in the development of peculiar sensorial characteristics of traditional fermented foods, through the production of enzymes and metabolites during the ripening period [1].

Fermented sausages belong to a vast group of products indicated as cured meats [2], and they are usually made with pork and/or beef meat stuffed in a case together with other ingredients (sugar, herbs and spices, starter cultures, etc.) and additives (curing salt, antioxidants, etc.), fermented by LAB and then submitted to drying and the maturation process. The quality of raw meat is essential to provide the organoleptic characteristics and the nutritional value to the final product. Therefore, the selection of animal breeds and their feeding system is essential [3]. Moreover, temperature and relative humidity control are essential to guarantee fermented sausages’ quality. During the ripening of raw meat sausages, the application of different preservation strategies (nitrate/nitrite), the growth of competitive microbiota, smoking, and drying are essential to prolong the shelf life [4].

**Figure 1:**
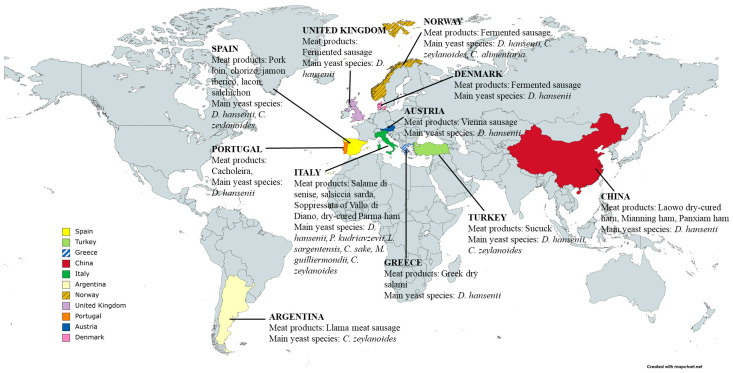
Fermented meats produced in different countries.

Like other fermented foods, fermented sausages represent a significant element of the cultural heritage of several countries. This is proven by the different receipts and the diversity of products diffused around the world and the large variety of products available on the market such as salami, salchichón, saussiçon sec, chorizo, summer sausage, and pepperoni [5] (Figure 1).

In Europe, it is possible to distinguish Northern and Mediterranean types of fermented sausages according to the rate of dryness, the ripening time, and the value of pH reached at the end of the production. Traditional fermented sausages are also produced in America, Argentina, Brazil, Uruguay, Asia, Africa, and China [6,7].

In contrast to Italian salami characterized by light fungal mycelium on the surface, Hungary and Holland salami and those produced in the U.S.A., specifically in the San Francisco–Oakland Bay area, have a surface covered by heavy fungal growth [8].

The global dry sausage market size was USD 4714 million in 2021 and is projected to reach USD 6931.07 million by 2031, at a Compound Annual Growth Rate of 3.9%.

The COVID-19 pandemic has increased the demand for food production and, in particular, it has determined high growth levels of the dry sausage market. This increased demand was mainly observed in America and Europe. Improvements in different aspects of sausage production, such as novel technologies and improved machinery, justify the optimistic forecasting [9]. In Italy, in 2022, an increase in the production of *salame* was observed, with a value of 125,300 tons (+42% compared to 2021). A positive contribution to the growth of the sector came both from internal consumption and the increase in foreign demand. A good result was achieved for the export—mainly EU countries such as France, Belgium, Sweden, Austria, and the Netherlands—despite the African swine fever disease and inflation [10].

LAB, yeasts, and filamentous fungi can be inoculated in the products to guide, accelerate, and standardize the fermentation process.

Filamentous fungi and yeasts can grow and colonize the fermented sausage surface [6], while yeasts have also been detected in the sausage’s interior [8]. Fungi positively participate in sausage production by the proteolysis and lipolysis reactions, providing aroma precursors [11]. The compounds resulting from glycolysis, proteolysis, lipolysis, and lipid oxidation (e.g., organic acids, peptides, amino acids, fatty acids) contribute to the characteristic flavor and texture of fermented meats [12]. Moreover, they exert technological properties, such as increasing pH by using products of lactic acid fermentation, preventing excessive drying of the product, and also facilitating sausage peeling, growth inhibition of undesired microorganisms, and detoxification properties [13,14]. On the other hand, some fungi have negative roles such as alteration of sensory characteristics and production of dangerous secondary metabolites [15,16].

Compared to the studies focused on LAB and filamentous fungi, those on yeasts have increased over the years. The recognition of the importance of yeasts in fermented sausages dates back to 1920 [17]; following this, in the early 2000s, different groups focused their research on yeasts, mainly belonging to *Debaryomyces* sp., and fermented sausages (see the review). Recently, research has extended interest towards various species of yeasts and not only to *Debaryomyces hansenii*, especially regarding their contribution to the flavor [18,19,20,21,22].

This review aims to give a concise overview of the literature on fungi in cured meats. It contributes to the existing literature by its novelty of considering the double role—positive and negative—played by filamentous fungi and yeasts, the global fermented sausage market size, the role and use of starters, and the starters mainly present in the worldwide market, as well as some recommendations that could be useful as starting points for novel research and technology transfer to the production.

## 2. Fungi and Fermented Sausages

Some fungal species originate from the environment, such as *Penicillium olsonii*, *Penicillium expansum*, *Penicillium viridicatum*, *Penicillium granulatum*, *Penicillium oxalicum*, *Penicillium commune*, *Aspergillus versicolor*, or *Aspergillus fischeri* [23]. Species belonging to the *Cladosporium*, *Mucor*, *Scopulariopsis*, or *Geotrichum* genera may also be sporadically encountered [24]. The most frequently isolated fungal species in fermented sausages are *Penicillium nalgiovense*, *Penicillium salami*, *Penicillium chrysogenum*, and *Penicillium nordicum* [24,25].

*D. hansenii* is the most frequently isolated yeast in fermented meats. Ramos-Moreno et al. [26] have recently reported worldwide dry-meat products from which *D. hansenii* has been isolated. Other yeast genera generally described on meat products are *Candida*, *Cryptococcus*, *Hansenula*, *Hypopichia*, *Kluyveromyces*, *Leucosporidium*, *Pichia*, *Rhodosporidium*, *Rhodotorula*, *Trichosporon*, *Torulospora*, and *Yarrowia* [8,27,28] (Figure 1). García-Béjar et al. [29] have recently isolated *Kazachstania servazzii*, *Yarrowia lipolitica*, *Saccharomyces cerevisiae*, *Wickerhamomyces anomalus*, *Torulaspora delbrueckii*, and *Meyerozyma guillermondii* from Spanish pork fermented sausages.

Yeasts and filamentous fungi are usually considered desirable in fermented sausages; however, they can have a dual role by either contributing to the quality and safety of the final product or being detrimental, causing spoilage and/or poisoning (toxigenic molds).

The quality and safety of fermented sausages are influenced by various factors other than fungi such as the raw materials, the environment, and the technology of production. Therefore, it seems essential to pay attention to the quality of the raw ingredients used but also to the hygienic conditions of the environment of production and the application of correct production technologies.

The organoleptic variability of the fermented sausages, such as the flavor, reflects the species and strain variability of yeasts and filamentous fungi naturally occurring or added as starters in the raw materials. The fungi, in fact, are able to metabolize them differently and interact among them in various ways.

Fermented sausages, like other fermented foods, can be considered as an ecosystem in which different microorganisms live in a community and their final qualities are the result of metabolic interactions among different yeasts; among yeasts and filamentous fungi; among different filamentous fungi; and among fungi and LAB.

Traditionally, fermentation takes place naturally, but it is advisable to use starter cultures able to guarantee the quality and safety of the final product. Considering the sausages are the result of a microbial community, in the selection process, it is useful to take into account not only the characteristics of a single microorganism but its behavior in the presence of other microorganisms concurring in the production process.

The negative and positive roles that filamentous fungi and yeasts can exert during the production of fermented sausages, their selection, and their use as starters are considered below.

### 2.1. Filamentous Fungi

Filamentous fungi associated with fermented sausages can have different origins. They can be part of the in-house mycobiota or deliberately inoculated, since surface molding is considered a positive event in several countries, e.g., Italy, Romania, Bulgaria, France, Hungary, Switzerland, Southern Germany, Spain, Austria, and Belgium.

The development of some fungal species on sausage casing is essential during drying and ripening since they exert a positive role; in fact, the mycelium contributes to the prevention of excessive drying of the final product, protects fat from oxidation, prevents rancidity, stabilizes the color through catalase activity, oxygen consumption, and protection against light, presents lipolytic and proteolytic activities, and makes sausage peeling easier.

Filamentous fungi extracellular proteases and lipases release amino acids and fatty acids which act as precursors of volatile compounds, having an effect on the aroma of the final product [30,31,32]. For instance, *P. chrysogenum* and *P. nalgiovense* showed lipolytic and proteolytic activities that produced flavor precursors and improved the texture [33]. *P. aurantiogriseum* improved the sensory properties of dry-fermented sausages, accelerating the production of degraded volatile compounds [34]. Other routes involved in aroma development are amino acid degradation, lactate oxidation, β-oxidation, and enzymatic activities. They are reviewed in depth by [35] and produce VOCs such as methyl ketones and aliphatic eight-carbon compounds. Moreover, a characteristic popcorn odor in mold-fermented sausages has been ascribed to 2-acetyl-1-pyrroline, which may be caused by the conversion of proline, often found in sausage collagen casings [35].

Another positive activity of the filamentous fungi mycelia concerns the protection of the product from oxygen and light resulting in an antioxidant effect on lipids, thus contrasting the rancidity, and stabilizing the color by catalase activity [36,37]. Moreover, mycelia act by preventing water loss, avoiding excessive dryness and resulting, also, in easy skin peeling [38,39].

Some filamentous fungi possess the ability to protect the sausage casing from the growth of spontaneous and undesired/toxigenic filamentous fungi [40], avoiding the problems reported below. This important role of protection is due to the competition for space and nutrients, to the production of substances such as proteins, peptides, and volatile compounds with antifungal properties [6,41].

*P. chrysogenum* can be considered a protective culture since it produces the antifungal protein PgAFP [42]. This filamentous fungus and the PgAFP were reported to be useful in decreasing the growth of toxigenic *Aspergillus flavus* and *Penicillium restrictum* in dry-fermented sausages [43], inhibiting cyclopiazonic acid production by *P. griseofulvum* during the ripening of dry-fermented sausages [44], decreasing the growth of ochratoxigenic *P. nordicum*, and reducing the concentration of OTA [45].

However, filamentous fungi can also have some negative effects related to the production of toxic compounds; moreover, fungal particles or conidia could cause respiratory diseases [46].

The main negative role of filamentous fungi is connected to the ability of some species to produce mycotoxins, secondary metabolites that are highly toxic when ingested [47]. Filamentous fungi considered atoxigenic are *P. nalgiovense* and *Penicillium salami*, while potentially toxigenic ones are *P. chrysogenum*, *Penicillium nordicum*, *P. olsonii*, *P. expansum*, *P. viridicatum*, *P. granulatum*, *P. oxalicum*, *P. commune*, *A. versicolor*, *A. fischeri* [25], *Aspergillus ochraceus* [48], and *Aspergillus carbonarius* [49].

In meat products, toxigenic filamentous fungi can in fact produce patulin, ochratoxin A (OTA), citrinin, and cyclopiazonic acid [50] and less commonly aflatoxins produced by *Aspergillus* spp. such as *A. versicolor* and *Aspergillus sydowii* [25,51,52]. Mycotoxins can be found on the casing but also inside the salami, since they migrate from the surface to the interior [53]. Therefore, removing the mold layer does not guarantee the safety of the product. The negative health effects are neurotoxicity, hepatoxicity, carcinogenicity, and nephrotoxicity [54].

Other than mycotoxins, some filamentous fungi can produce antibiotics such as penicillin and negapillin [47,55], determining another health risk factor.

Another undesired effect is contaminant filamentous fungi that determine spoilage of the product growing on the casing as black spots or green and grey spots/covering (*Cladosporium oxysporum* and *Penicillium* spp.) [16,25]. Spoilage has also been reported as superficial white spots and white thin fibers in the interior [56]. Usually, these defects are not linked with unpleasant odors but they render the product unacceptable to the consumers anyway. Some filamentous fungi may also spoil the product due to the production of off-odors and unpleasant taste [35,57].

In conclusion, to avoid quality and safety problems of fermented sausages due to spoilage and toxigenic filamentous fungi, it is advisable to pay attention to the good quality of the ingredients used, the hygienic condition of the site of production to avoid unwanted contamination and, more importantly, to make use of starter cultures specifically selected from toxicological and technological points of view. The role and effect of species and strains of filamentous fungi on the final product have to be considered in a global view; therefore, their evaluation has to take into account multiple factors including the raw material, the parameters of ripening, and the possible metabolic interaction among the various components of the microbiota involved in the production process.

### 2.2. Yeasts

Usually, yeasts are known for their positive role in transforming raw materials into foods and beverages such as bread, wine, and beer through fermentation. Actually, they can cause food spoilage, especially when they reach concentrations of 10^5^–10^6^ CFU/g [58].

Yeasts, similarly to filamentous fungi, have an antioxidant effect that protects the product against oxygen and avoids excessive product drying, regulating the humidity fluctuation [59]. Moreover, they can delay rancidity thanks to their oxygen-scavenging and lipolytic activities [60,61]. For instance, the development of *D. hansenii* on the surface of fermented sausages is essential to regulate water release during ripening and to reduce hardness and chewiness in sausages. In addition, lipid oxidation inhibition in yeast-inoculated sausages results in a reduction in TBARS (Thiobarbituric Acid Reactive Substance) values. This reduction was associated with descriptors such as white fat and fatty tastes, and fruity odor notes [62].

Yeasts also act by stabilizing color and affecting aroma [63]. They influence the flavor of fermented sausages by proteolytic and lipolytic activities [64] and by the generation of several volatile organic compounds (VOCs) such as ethyl 2-methylpropanoate, ethyl 2-methylbutanoate, ethyl 3-methylbutanoate, 4-methylphenol, ethyl benzoate, benzothiazole, 2,4-decadienal, methyleugenol, and γ-nanolactone [65]. Actually, ester compounds are mainly produced by coagulase-negative staphylococci (CNS); however, LAB, yeasts, and filamentous fungi also contribute to their production and to the fermented sausage aroma [66]. Since *D. hansenii* dominates in fermented sausages, the majority of studies are focused on this species [67,68]. However, it is still not clear how *D. hansenii* influences the organoleptic properties and appearance of sausages and dry-meat products. Its impact on the characteristics of these products could possibly be due to its ability to ferment different sugars, its degradation of peroxides and amino acids, and its lipolytic activity [26,64]. On the other hand, some authors have reported proteolytic activity of *Saccharomyces cerevisiae* isolated from Italian salami [69], lipolytic activity of *Yarrowia lipolytica* [70], proteolytic and lipolytic activities and free fatty acid release of *Y. lipolytica* [71], and VOC production by *Candida utilis* in dry-fermented sausages [72].

Recent research has contributed to increasing the literature on yeasts and extending the knowledge about the behavior of different yeast species in fermented sausages especially regarding their contribution to the flavor. Strains of *S. cerevisiae*, *Kazachstania exigua*, *Kazachstania bulderi*, and *C. zeylanoides* showed lipolytic activity and produced esters [18]. *P. kudriavzevii* is proposed as a starter in reduced-salt dry sausages due to its positive effect on the aroma [19]; in addition, potential enhancers of aroma are strains of *T. delbrueckii*, *P. kudriavzevii*, and *C. zeylanoides* tested in a model system [20].

Despite the newest studies and knowledge about the positive role of different yeast species in fermented sausages, they are not included in the commercial starter available on the market besides the approved *D. hansenii* [73].

Another factor to take into consideration is that the modification of ingredients and recipes can affect the final product differently. For instance, inoculation of *D. hansenii* on dry-fermented sausages manufactured with reduced ingoing amounts of nitrite and nitrate has been very recently evaluated, focusing on VOC production. Different *D. hansenii* strains showed abilities to develop in media containing nitrate and nitrite; these substances can or cannot affect the production of VOCs, and this was found to be strain-dependent [74]. Another study on the flavor reported that ester production is associated with yeast concentration and it is strongly strain-dependent [75]. The effect of nitrite and nitrate reduction in fermented sausages was also observed as a reduction in lipid oxidation and generation of derived volatiles [67].

Recently, some studies highlighted important roles of *D. hansenii* in fermented sausages other than the role of reducing lipid oxidation, enhancing the content of volatile compounds, and preventing nitrite oxidation: (i) contribution to masking unwanted fat odors [63,76]; (ii) reduction of meat mutagens and biogenic amines accumulation [77]; (iii) control of *Listeria monocytogenes* [78].

Yeasts can be considered as biocontrol cultures due to their important positive roles affecting the safety of the final product. In fact, they break peroxides and consume oxygen, making it unavailable to pathogenic microorganisms [64]. Yeasts can have a biocontrol role against toxigenic filamentous fungi [79] by different mechanisms such as microorganism competition [80], production of killer toxins [81], production of volatile antifungal compounds [6], and mycotoxin adsorption [82,83].

*D. hansenii* was able to control spontaneous filamentous fungi development on fermented Sardinian sausages [84]. *Hyphopichia burtonii*, *D. hansenii*, *Saccharomycopsis fibuligera*, and *C. zeylanoides* showed inhibition activity against *P. nordicum*, *P. verrucosum*, and *A. ochraceus* [45,85,86]. García-Béjar et al. [29] have recently reported that strains of *Candida metapsilopsis*, *D. hansenii*, *Kazachstania servazzii*, *M. guillermondii*, *S. cerevisiae*, and *W. anomalus* did not show activity against *Aspergillus parasiticus*, *Fusarium graminearum*, and *Penicillium crustrosum*; all of them except for the *K. servazzii* strain showed inhibition of *F. graminearum*, while two strains of *D. hansenii* and the *K. servazzii* strain inhibited *P. crustorum*. Recently, it has been reported that *D. hansenii* affected the growth of *Aspergillus westerdijkiae* and its OTA production [87]. Endogenous yeasts of Portuguese dry sausages were able to control *P. nordicum* and *A. westerdijkiae* growth and OTA production [88]. Interestingly, the same authors have reported that the co-existence of endogenous yeasts and commercial starter culture composed of LAB, CNS, and *D. hansenii* stimulated OTA production by the two filamentous fungi. Yeasts can antagonize the growth of undesired bacteria; as an example, *D. hansenii* inhibited *Staphylococcus aureus* growth in fermented sausages [89].

The negative role of the yeasts is essentially related to the spoilage of the fermented sausages. Typical yeast spoilage of processed meats results in the production of off-odors and discolorations [58]. In a meat model substrate, sensorial changes denoting spoilage, such as slime formation, discoloration, and off-flavors, appeared when *Candida zeylanoides*, *D. hansenii*, *Pichia guilliermondii* (syn. *M. guilliermondii*), and *Candida sake* reached levels of 10^5^–10^6^ CFU/g [63].

Another negative role of yeasts concerns their potential ability to neutralize the sodium nitrite used as a preservative by the production of acetaldehyde [58,70,90]. This aspect affects the safety of the product due to the important role of sodium nitrite against *Clostridium botulinum*.

Examining the literature about yeasts and their effect on sensory characteristics, controversial results are often observed among different authors [26,64]. For instance, no color improvement was reported by Cano-García et al. [91], while the opposite results were reported by Corral et al. [76]. Moreover, a correspondence between yeast activity and positive sensory effects has not always been observed. No sensory effects have been reported by some authors [72,75,92], while positive sensory effects have been reported by others [93,94]. This controversy could be due to the different types of meat and manufacturing used but also to the microbial communities with their metabolic interactions varying among the products considered.

In conclusion, the characteristics of the final product given by the yeasts can vary according to multiple factors such as meat ingredients, preservatives and their modifications, technological parameters, ripening time, species of yeasts and strain variability, use of starters, and yeast interaction with the other microbiota inhabiting the same environment.

As regards the obtainment of novel starters belonging to species different from *D. hansenii*, in some cases, different yeast species are considered potential aroma producers since they were tested in a model system; therefore, novel and more studies in real fermented sausages are welcomed as well as the proposal and approval of new starters in the market.

## 3. Yeasts and Filamentous Fungi as Detoxification Tools

Yeasts and filamentous fungi have been suggested as a promising strategy to reduce mycotoxin contamination in fermented foods including meat products [14] (Table 1). Three mechanisms have been proposed: (i) inhibition of mycotoxin production; (ii) biodegradation of mycotoxins; and (iii) detoxification by adsorption on the cell wall. Yeasts can release metabolites that inhibit mycotoxin production, for instance, 2-phenylethanol released by *W. anomalus*. This compound inhibits spore germination and aflatoxin B1 (AFB1) biosynthesis by *A. flavus* through the downregulation of some aflatoxin biosynthesis genes such as *aflC* (polyketide synthase, an early gene in the AF pathway), *aflR* (a positive aflatoxin pathway regulator), *aflS* (transcription enhancer), *aflO* (O-methyltransferase B), and *aflK* (versicolorin B synthase). In addition, it is also able to reduce the growth of this fungus, altering the expression of the *MYST1*, *MYST2*, *MYST3*, *gcn5*, *hdaA*, and *rpdA* genes [95]. As stated above, *D. hansenii* was able to inhibit OTA biosynthesis of *A. westerdijkiae* at the level of transcription [96], and *S. cerevisiae* inhibited the growth of Aspergilli and the OTA production of *A. carbonarius* and *A. ochraceus* by downregulating the *pks* (polyketide synthase) biosynthetic gene [97]. Some yeasts such as *S. cerevisiae*, *Geotrichum fermentans*, *K. marxianus*, *M. pulcherrima*, *Hanseniaspora*, *Trichosporon*, *Rhodotorula*, and *Cryptococcus* spp. can degrade mycotoxins using mechanisms not fully understood. Some authors proposed that some yeast species release carboxypeptidases, which transform OTA into its less toxic derivative ochratoxin -α (OT-α) [98]. For instance, *Trichosporon*, *Rhodotorula*, and *Cryptococcus* spp. were able to split the amide bond of the OTA molecule and release non-toxic OT-α [99]. *Aspergillus niger* GX312, *A. japonicus* AX35, and *A. carbonarius* SA332 (a weak OTA producer) can convert OTA to OT-α, which in turn is converted to an unknown compound [99]. *Pichia caraibica*, *M. pulcherrima*, *P. ohmeri*, *R. kratochvilovae*, *S. cerevisiae*, and *Sporobolomyces* spp. show patulin-degrading abilities [100,101].

Yeasts are key players in mycotoxin detoxification by adsorption of toxins on their cell walls. Yeast species are characterized by a diverse cell wall composition in terms of β-glucans, glucomannans, and mannan-oligosaccharides, which are the main compounds responsible for mycotoxin adsorption [102]. Yiannikouris et al. [102] showed that the content of β-glucans and its three-dimensional arrangement in the cell wall affects mycotoxin adsorption. Moreover, some studies revealed that the structural integrity of the yeast cell wall and non-viable cells are generally more effective in their adsorption capacity [103,104].

The main yeast species involved in this process are *S. cerevisiae*, *C. laurentii* (reclassified *Criptococcus lautentii*), *Kloeckera* spp., *Rhodotorula glutinis*, *Saccharomycodes ludwigii* (reclassified *S. kudriavzevii*), *Schizosaccharomyces pombe*, *T. delbrueckii*, and the most studied *D. hansenii*. The amount of removed toxin depends on the species and the strains, the toxin and microorganism concentrations, the cell wall composition, and the total amount of cell wall [105].

Several efforts should be made in this field, especially to better understand the molecular mechanisms underlying mycotoxin biodegradation, unwanted effects on the quality of the detoxified products, the transcriptional and post-transcriptional control of toxin production, and the inheritance and stability of detoxification-related traits of yeasts. This kind of study should be useful to develop new pipelines of strain selection and eventually mixed starter cultures of yeasts and filamentous fungi to exploit the synergism in mycotoxin degradation.

## 4. Yeasts and Filamentous Fungi as Starter Cultures

Starter cultures are microorganisms able to outcompete the autochthonous ones and possess useful traits leading to the manufacture of fermented meat products improved in quality, safety, and shelf life [106].

Since the 1970s, *D. hansenii* and *P. nalgiovense* have been indicated as starter cultures for meat products [35,60]. Several studies have proposed the use of starter cultures containing *D. hansenii* in combination with other microorganisms, in particular LAB and staphylococci. In fact, the inoculation of dry-fermented sausages with *Debaryomyces* spp., LAB, and staphylococci microorganisms had a positive effect on the final flavor and sensory qualities of the product through the production of ethyl esters [26]. In this context, it should be remarked that the number of yeasts used in the starter culture must be optimized, since too many yeasts may mask some positive effects.

Filamentous fungi and yeasts used as starter cultures should show some specific characteristics: (i) acid production; (ii) positive contribution to the product color and aroma; (iii) improved safety and reduced hygienic and toxicological risks (e.g., absence of mycotoxin and antibiotic production, and absence of pathogenicity); (iv) growth at 15 °C, tolerance toward low pH values, low water activity, and high salt concentrations; (v) proteolytic and lipolytic activities; (vi) catalase positivity; (vii) absence of cellulolytic activity; (viii) white mycelium. Another important factor is the interaction of selected starter strains in mixed cultures, as well as the competitive behavior, viability, and survival; antagonism against pathogens and spoilage microbes; degradation of anti-nutritive factors; detoxification; and eventually probiotic traits [35,106,107] (Table 2).

Yeast starters can be inoculated on the casing by dipping or spraying methods or in the interior at a 4–6 log CFU/g concentration. On the contrary, filamentous fungi starters are usually inoculated on the surface by the dipping method at spore concentrations ranging from 3 to 4 log spores/cm^2^ [106].

Limited information about the worldwide law regulation on starter cultures for processed meats is available. According to the Ministero della Sanità [108], *D. hansenii*, *P. nalgiovense*, and *P. chrysogenum* are authorized to be used as starter cultures in fermented meat products. Since the Italian regulation has adopted EU regulation, it is very likely that the above-reported species are allowed to be used in other European countries.

Different authors have reported the selection of yeasts to improve the quality of fermented sausages [109,110]. Other studies have evaluated commercial starters alone or in combination with indigenous cultures in the production of processed meats [6,88,111].

Currently, starters of yeasts and molds are available on the market, and Table 3 reports a selection of commercial starters along with companies’ claims.

The use of fungal starters, like bacterial ones, is advisable both for quality and safety aspects. Despite the difference in the raw material used, the application of commercial starters could lead to a standardization of the fermented sausages produced. A possible method to maintain variability among the productions is to isolate and select autochthonous fungi strains for starter use that could give specificity to the production.

**Table 2:** The main effects of different inoculated yeasts in dry-cured meat products.

Inoculated Yeast	Main Effects	Meat Product	Country	References
*Debaryomyces hansenii*, *Candida deformans*, and *Candida zeylanoides*	VOC increase	Dry-cured ham (Lacon)	Spain	[105,106,107,108,109]
*D. hansenii*	VOC increase, improved appearance and texture, slight toasted flavor, high acceptability	Dry-cured ham	Spain	[33]
	VOC increase	Dry-cured loin	Spain	[112]
	VOC increase, antioxidant effect	Dry-fermented sausages	Spain	[64]
	Proteolytic activity, amino acid release, ammonia decrease	Dry-fermented sausages	Spain	[113]
	Antioxidant effect, VOC production (acid, sulfur, and esters)	Dry-fermented sausages	Spain	[75]
	Increased lipolysis and antioxidant effects, increased VOCs derived from amino acids and ester compounds, positive sensory effect, high consumer preference, aroma increased	Dry-fermented sausages	Spain	[94,114]
	VOC increase	Dry-fermented sausages	Spain	[67,115]
*D. hansenii*, *Yarrowia lipolytica*, *Trichosporon mucoides*	Lipolytic activity	Dry-fermented sausages	Spain	[92]
*D. hansenii* and *Candida utilis*	VOC increase	Dry-fermented sausages	Denmark	[72]
*D. hansenii*, *Y. lipolytica*	a_w_ decrease, lipolytic activity, free fatty acid release, proteolytic activity, VOC increase, improved appearance, increased consumer acceptance	Dry-fermented sausages	Italy	[71,116]

**Table 3:** Fungi starter cultures present on the market and their role in fermented sausage production.

Product	Specie	Main Features and Benefits	References
Lallemand Meat Surface PS521	*Penicillium nalgiovense*	Mediterranean-style flavor, uniform white cover, inhibition of undesirable mold growth, prevention of oxidation	“https://testek.ca/en/produit/lallemand-meat-surface-ps521-10g/ (accessed on 14 April 2024)”
PNC110	*P. nalgiovensis*, *Penicillium candidum*	Mediterranean-style flavor, white and downy covering	“https://www.ingredientsnetwork.com/47/resourcefile/12/21/44/LSC%20-%20Meat%20portfolio_EN_042021.pdf (accessed on 14 April 2024)”
PC PSM2	*P. candidum*	Protection against *Mucor*, white and downy covering	
PC TN	*P. candidum*	White and downy covering, production of a traditional Iberian-style flavor	
Flavor Start D306	*Lactobacillus Staphylococcus Geotrichum*	Unique and complex flavor, prevention of growth of some pathogens	“https://specialty-cultures.lallemand.com/wp-content/uploads/2017/10/LSC-MEAT-SnR-Culture-Portfolio-June-2017.pdf (accessed on 14 April 2024)”
Mold 600	*P. nalgiovensis*	Strong suppression of wild flora, positive effect on the drying process, uniform white coverage, mushroom flavor	“https://hjemmeriet.com/da/ChrHansen/Brochures/Meat%20manual_UK.pdf (accessed on 14 April 2024)”
Mold 500	*Debaryomyces hansenii*, *P. nalgiovense*	Moderate suppression of indigenous flora, short coverage, Italian-style flavor	
Mold 800	*P. candidum*, *P. nalgiovense*	Strong suppression of indigenous flora, medium to fluffy coverage, Camembert aroma/mushroom flavor/scent of moss	
SM-182	*Latilactobacillus sakei*, *Staphylococcus carnosus*, *D. hansenii*	Mediterranean flavor	
SM-194	*Pediococcus pentosaceous*, *L. sakei*, *S. carnosus*, *Staphylococcus xylosus*, *D. hansenii*	Mediterranean flavor, stable color	
Startec TCSM1 Muffa	*P. nalgiovense*	Atoxigenic, fast white/grey/ivory coverage, proteolytic and lipolytic, inhibition of undesirable microorganisms	“http://www.tecalsrl.com/page.php?lang=eng&idpagina=17 (accessed on 14 April 2024)”

## 5. Roles of Typical Fungi in Fermented Sausage Production

This paragraph reports the role of two typical fungi widely used to produce fermented sausages: *D. hansenii* and *P. nalgiovense*.

The contribution of *D. hansenii* to the quality of meat products is usually associated with its capacity to ferment different sugars and degrade peroxides and amino acids. Consequently, these activities produce acetaldehyde, ethyl acetate, or alcohols, as well as a variety of other volatile compounds. The production of volatile compounds by *D. hansenii* is one of the most important contributors to flavor development during the ripening process in dry-fermented meat products. *D. hansenii* modifies the levels of volatile and aromatic compounds by increasing esters (essential contributors to the aroma of meat products) and alcohol metabolites in comparison to the non-inoculated sausages. In addition, *D. hansenii* metabolizes lactic acid produced by LAB, contributing to the final organoleptic characteristics of the product. Moreover, this yeast may hydrolyze pork fat during the processing of fermented meat products. The lipase activity is not affected by NaCl (most probably due to the salt-tolerant character of this yeast), and it is still significant at pH 4.7, although it is highly dependent on the time and ripening conditions. The introduction of *D. hansenii* as a starter culture could contribute to flavor, color, and texture development during the ripening of fermented meat. The different effect reported in the literature depends on the selected strain, the quantity of the inoculated yeast, the type of sausage, the manufacturing process, and even the presence of other starters (for a review on *D. hanseni* in meat products, see 26).

*P. nalgiovense* is inoculated on the surface of sausages as a suspension of asexual spores and develops a typical thin layer of mycelium. During the maturation/ripening process, it creates a microclimate on the surface that prevents moisture loss. Moreover, it protects the sausage from light and oxygen, prevents rancidity, and acts as a biocontrol agent against undesired microorganisms. An important part of the maturation stage is the proteolytic and lipolytic activities that contribute to the texture and flavor of the mature product [117].

## 6. Gaps and Future Recommendations

Gaps and limitations in the research have emerged after critically reviewing the worldwide literature on fermented sausages and fungi. Some recommendations that could be useful as a starting point for novel research and technology transfer for sausage production are reported in the following.

Recently, attention has been given to the bacterial communities of fermented meats by a High-Throughput Amplicon Sequencing Method [118,119]. This methodology has recently been used to study the fungal communities involved in this type of production [21,22]. However, more studies are welcomed since the improvement of these products is achieved when considering the fermented sausage as an ecosystem in which different microorganisms concur for its production. The majority of studies were focused on describing the yeast composition of fermented sausages or on *D. hansenii* and its effects on production. Only recently have other yeast species isolated from fermented sausages been studied for useful biotechnological properties [18,19,20,29,88]. Similar future studies are warranted to select autochthonous yeast species different from the most studied and used starter cultures of *D. hansenii*. This will allow for the valorization of local products and for an excess of uniformity in the production to be avoided. Moreover, another aspect to take into account is the knowledge of the metabolism of the yeast under study and its behavior towards other microorganisms; this could be useful, for example, when considering the relation between yeasts and toxigenic filamentous fungi. Therefore, studies should consider not only a singular microorganism and its role in sausage production but also its interactions with the population of the sausage microbial community. Future studies on yeasts are also welcomed to widen the current species of *D. hansenii* approved and available on the market as starter cultures with the aim of diversifying production.

In conclusion, a framework to follow for future research could be to consider the fungal communities and the driving interactions among species, study novel species of fungi, and technological transfer to novel starter cultures.

## Figures and Tables

**Table 1:** Main yeast and filamentous fungi species involved in mycotoxin decontamination. Modified from Piotrowska et al. [14].

Mechanism	Species	Targeted Mycotoxins
Biodegradation	*Candida guilliermondii*, *Candida parapsilosis*, *Candida utilis*, *Kodameae ohmeri*, *Komagataella phaffi*, *Metschnikowia guilliermondii*, *Metschnikowia pulcherrima*, *Pichia carribica*, *Rhodosporidium kratochvilovae*, *Rhodotorula mucilaginosa*, *Saccharomyces pastorianus*, *Yarrowia lipolytica*, *Kazachstania servazzii*, *Kloeckera apiculate*, *Trichosporon mycotoxinivorans*; *Aspergillus niger*, *Byssochlamys nivea*, *Clonostachys rosea*, *Rhizopus oryzae*, *Rhizopus stolonifer*, *Rhizopus microsporus*, *Rhizopus homothallicus*, *Trichoderma reesei*, *Cladosporium uredinicola*, *Aspergillus carbonarius*, *Aspergillus fumigatus*, *Aspergillus clavatus*, *Aspergillus ochraceus*, *Aspergillus versicolor*, *Aspergillus wentii*, *Penicillium aurantiogriseum*, *Penicillium spinulosum*, *Pleurotus ostreatus*, *Aureobasidium pullulans*	PAT, ZEN, FB1, OTA, DON, AFB1
Adsorption	*Candida utilis*, *Saccharomyces cerevisiae*, *S. pastorianus*, *Candida laurentii*, *Kloeckera* spp., *Rhodotorula glutinis*, *Saccharomycodes ludwigii*, *Schizosaccharomyces pombe*, *Torulaspora delbrueckii*	ZEN, OTA, AFB1, PAT, DON, AOH, AME

PAT: patulin; ZEN: zearalenone; FB1: fumosin B1; OTA: ochratoxin; DON: deoxynivalenol; AFB1: aflatoxin B1; AOH: alternariol; AME: alternariol monomethyl ether.

## Data Availability

No new data were created or analyzed in this study. Data sharing is not applicable to this article.
